# An MRI-based feasibility study of unilateral percutaneous vertebroplasty

**DOI:** 10.1186/s12891-015-0619-x

**Published:** 2015-07-10

**Authors:** Haijun Li, Lei Yang, Jian Tang, Dawei Ge, Hao Xie, Jinhua Chen, Lipeng Yu, Haifeng Wei, Weizhong Tian, Tao Sui, Xiaojian Cao

**Affiliations:** Department of Orthopedics, the First Affiliated Hospital of Nanjing Medical University, 300 Guangzhou Road, Nanjing, 210029 Jiangsu China; Department of Radiology, Taizhou People’s Hospital affiliated to Nantong University, Taizhou, Jiangsu Province China; Department of Orthopedics, the Second Affiliated Hospital of Nanjing Medical University, Nanjing, Jiangsu Province China

**Keywords:** Pedicle, MRI, Unilateral PVP, Puncture, Radiation exposure

## Abstract

**Background:**

Percutaneous vertebroplasty (PVP) has been demonstrated to be effective in the treatment of osteoporotic fracture. The bilateral pedicular approach is the most frequently used method. However, unilateral PVP is becoming increasingly more attractive for surgeons because of its numerous benefits, including lower radiation exposure, less tissue injury, and less bone cement leakage. The purpose of this study was to investigate the anatomical feasibility of unilateral PVP by exploring the differences in the puncture success rate of the unilateral pedicular approach among different lumbar segments, between men and women, and between the left and right sides.

**Methods:**

Punctures were simulated on magnetic resonance imaging scans of 200 patients (100 men, 100 women) at a maximum angle via a pedicular approach. The distance between the entry point and the midline of the vertebral body, the maximum puncture angle, the puncture success value, and the puncture success rate were measured and compared among different lumbar levels, between the two sexes, and between the left and right sides.

**Results:**

The maximum puncture distance between the entry point and the midline gradually increased from L1 to L5, and the maximum puncture angle showed the same tendency from L1 to L5. The puncture success values for L3 and L4 were higher than those for the other lumbar levels (L1, 31.53 ± 34.45; L2, 42.15 ± 28.06; L3, 56.21 ± 18.30; L4, 56.20 ± 12.93; and L5, 48.01 ± 6.88). The puncture success rates varied from 69.5 to 98.0 % among the different lumbar levels; L3 and L4 were the two highest (L3, 95.5 %; L4, 98.0 %). There were significant differences in these measurements between men and women and between the left and right sides.

**Conclusions:**

PVP with the unilateral puncture approach appears more likely to succeed at L3 to L5 than at L1 and L2. The unilateral approach might be more suitable for men than women at levels other than L5. Additionally, the left pedicular approach might be optimal for unilateral PVP procedures.

## Background

Percutaneous vertebroplasty (PVP) has been widely used to treat osteoporotic fractures. This technique has been shown to be an effective treatment method with numerous advantages, including limited trauma, brief patient immobilization, few complications, and reliable safety [1–8].

Frequently used PVP approaches include the pedicular, parapedicular, anterolateral, and posterolateral approaches. PVP using the bilateral pedicular approach aided by a C-arm is the most frequently used method. Surgery via the unilateral pedicular approach reportedly achieves a clinical effect identical to that of the traditional bipedicular approach [9–13], but with lower radiation exposure, less tissue injury, less bone cement leakage, and a shorter operation time. However, unilateral puncture failure is not rare, and a biomechanical imbalance could be caused by uneven bone cement distribution. A fractured vertebra can reportedly be supported with stable biomechanics by unilateral PVP only if the bone cement distribution exceeds the midline [14]. Although enlarging the puncture angle may solve this problem, it is associated with the risk of cortical perforation and spinal cord and nerve root injury. Accordingly, whether PVP should be conducted via a unilateral or bilateral approach remains a matter of debate [12, 14, 15].

Because of the anatomical distinctions among different lumbar levels and between the two sexes, the difficulty of unilateral puncture is not identical in all cases. It appears to be more likely to succeed for some lumbar levels; however, it is risky for other levels, and a bilateral approach might be more appropriate [9]. Basic anatomical research on the differences in the success rates among the levels and between the sexes has rarely been reported. In this study, we explored the differences in the success rates of unilateral PVP among different lumbar levels, between the sexes, and between the right and left sides based on magnetic resonance imaging (MRI) to provide reference points for selection of the optimal puncture approach in PVP.

## Methods

This study was approved by the ethics committee of the First Affiliated Hospital of Nanjing Medical University, and written informed consent was obtained from all patients. The MRI scans of 200 patients (100 men, 100 women) were collected from 15 November 2013 to 5 December 2013 from outpatients with lower back pain, regardless of their lower radicular symptoms. The MRI scans were obtained with a 3-Tesla Siemens coil (Verio 3.0 T; Siemens AG, Erlangen, Germany) in the course of regular patient care. We included adults aged 18 to 70 years at the time of MRI. The following exclusion criteria were used: developmental abnormalities, including cretinism, dwarfism, and gigantism; vertebral abnormalities, including lumbar sacralization, sacral lumbarization, hemivertebrae, wedged vertebrae, butterfly vertebrae, vertebral fusion, scoliosis, lordosis, kyphosis, and spinal bifida; and a history of lumbar surgery. The images were analyzed with SkyViewPACS V3.3.1.5 (Yangtze River Ruiheng Software, Ltd., Nanjing, China) with a length precision of 0.1 mm and an angle precision of 0.1 °. Two spinal neurosurgeons with more than 10 years of experience performed the measurements. All images were numbered before the analyses, after anonymization of the patients’ information. All observers were blinded to the age and sex of the patients.

**Fig. 1:**
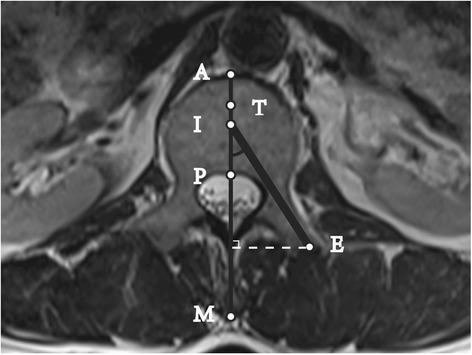
Cross-sectional MRI scan of lumbar pedicle: Successful puncture at the Max-angle. Line M: Anteroposterior midline of the vertebrae; A: the intersection point of Line M and the vertebral anterior edge; P: the intersection point of Line M and the vertebral posterior edge; T: the anterior trisection point of Line AP; ∠EIP: Puncture Max-angle; Line EI was used to simulate the puncture device which was 3.5 mm in diameter; the inner edge of Line EI was tangential to the medial wall of the pedicle, and the outer edge of Line EI was tangential to the lateral wall. In Fig. 1, the Puncture Success Value = 100*AI/AP > 34, and it was considered a successful puncture

**Fig. 2:**
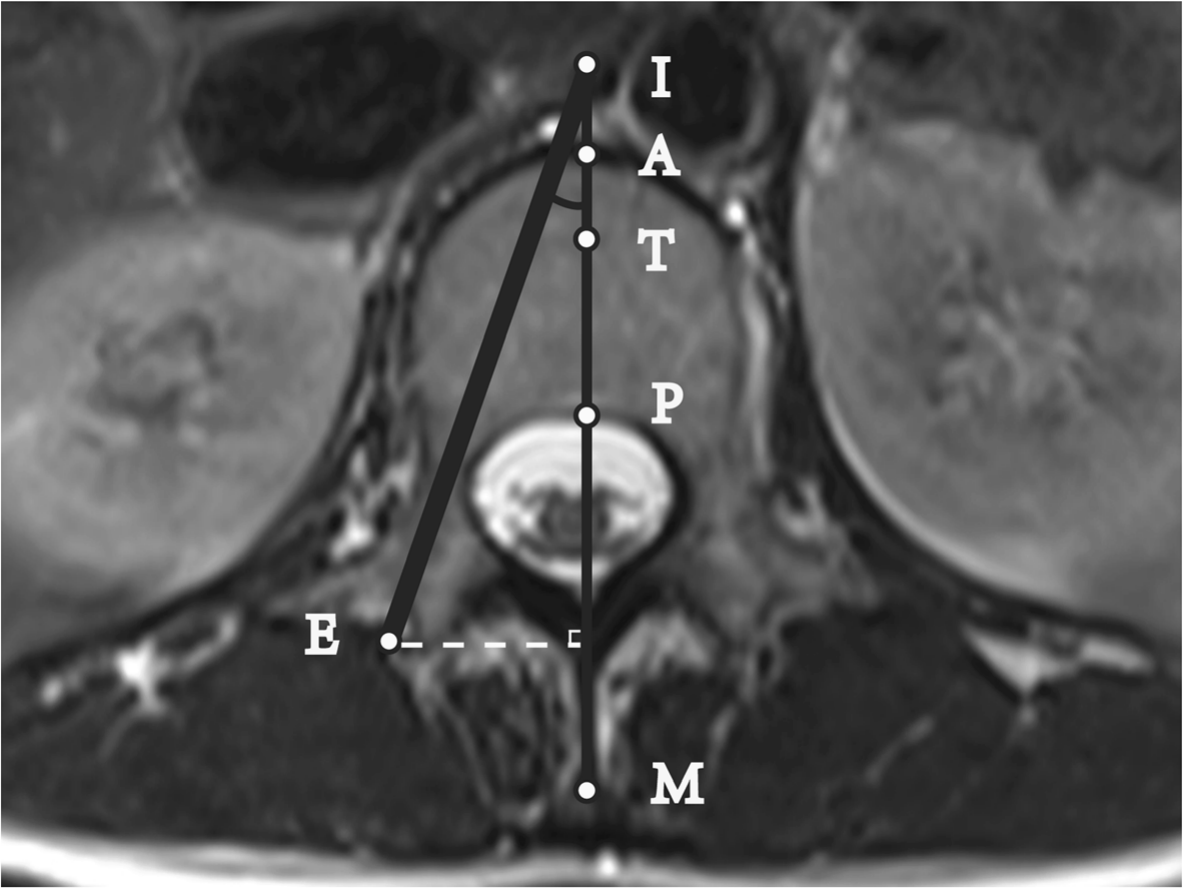
Cross-sectional MRI scan of lumbar pedicle: Unsuccessful puncture at the Max-angle. Line M: Anteroposterior midline of the vertebra; A: the intersection point of Line M and the vertebral anterior edge; P: the intersection point of Line M and the vertebral posterior edge; T: the anterior trisection point of Line AP; ∠EIP: Puncture Max-angle; Line EI was used to simulate the puncture device, which was 3.5 mm in diameter; the inner edge of Line EI was tangential to the medial wall of the pedicle, and the outer edge of Line EI was tangential to the lateral wall. In Fig. 2, point I was on the extension line of AP, and AI was defined as a negative value. The Puncture Success Value = 100*AI/AP < 34, and the puncture was considered a failure

All vertebral bodies were scanned through the pedicle and parallel to the upper vertebral endplate from L1 to L5. Three T2-weighted tomographic images were obtained for each segment. We selected the tomographic image with the largest pedicle width for precise measurements (Figs. 1 and 2). The caliper and protractor functions of the SkyViewPACS software were used to measure the distance and angle, respectively.

### Puncture simulation

The general puncture device was 3.5 mm in diameter, and we used a line 3.5 mm in diameter to simulate the puncture process (Line EI in Fig. 1).

As shown in Fig. 1, Line EI was inserted in the pedicle at the maximum angle required to meet the following conditions: the inner edge of Line EI was tangential to the medial wall of the pedicle, and the outer edge of Line EI was tangential to the lateral wall. Point I was the intersection point of Line EI and Line M, and Point E was the entry point (at the end of the pedicle, not at the skin). If the extension of Line EI crossed the iliac crest on the cross-sectional MRI scan, the puncture could not be performed at the maximum angle. Thus, the direction of the puncture needed to be adjusted to be tangential to the medial wall of the iliac crest.

### Measurements

The measurements included the following four indicators:

1. Maximum puncture distance: the vertical distance from Point E to Line M.
2. Maximum puncture angle: the value of ∠EIP.
3. Puncture success value: based on the ratio of AI to AP and calculated as (AI *100) / AP. As shown in Fig. 1, when Point I was on Line AP, the value of AI was positive. Instead, when Point I was on the extension line of AP, then the value of AI was negative (see Fig. 2).
4. Puncture success rate: Line AP was trisected, and Point T was the anterior trisection point. Generally, when Point I was anterior to Point T, the puncture was considered a failure. A successful puncture had a puncture success value of ≥34 [12, 16].

The following six success rates were assessed:

1. Left success rate
2. Right success rate
3. Bilateral success rate (puncture success values of both sides were ≥34 for identical vertebrae)
4. Either-side success rate (puncture success value of either side was ≥34)
5. Female success rate (e.g., if a puncture was performed through both sides at the L1 level, then there were 200 punctures among 100 women; if 84 of the 200 punctures were considered successful, then the female success rate = 84/200)
6. Male success rate (calculated as for the female success rate)

### Statistical analysis

The data are presented as mean ± standard deviation. The data were analyzed using SPSS 20.0 (IBM Corp., Armonk, NY, USA). Differences in the measured data between the left and right sides were analyzed at each vertebral segment with a paired t-test. Differences in the measurements between the sexes were examined by independent t-tests. One-factor analysis of variance in conjunction with the least significant difference test was used to examine the differences among the five segments. The chi-squared test was used to analyze the enumerated data. The statistical analyses were two-sided, and P < 0.05 was considered statistically significant.

## Results

The data were tested for Gaussian distributions. The mean ± standard deviation of the maximum puncture distance and maximum puncture angle are shown in Tables 1 and 2; the puncture success value and puncture success rate are shown in Tables 3 and 4. For the distance measurements, the interobserver error averaged ±1.17 mm, and the intraobserver error averaged ±1.01 mm. For the angle measurements, the interobserver error averaged ±1.44 °, and the intraobserver error averaged ±1.12 °.

**Table 1:** Puncture Max-distance (mm)

	L1	L2	L3	L4	L5
Left	22.07 ± 2.79	23.39 ± 2.77	25.79 ± 3.14	26.66 ± 3.31	29.93 ± 3.96
Right	21.85 ± 2.62	23.30 ± 2.95	25.18 ± 2.87*	26.06 ± 2.82*	29.96 ± 4.08
Female	20.88 ± 2.16	22.02 ± 2.35	24.42 ± 2.50	27.22 ± 3.16	29.75 ± 4017
Male	23.04 ± 2.77**	24.67 ± 2.70**	26.55 ± 3.12**	25.50 ± 2.77**	30.14 ± 3.86**
Mean	21.96 ± 2.70	23.35 ± 2.86	25.48 ± 3.02	26.36 ± 3.09	29.94 ± 4.02

*Compared with the left,side *P* < 0.05; ** Compared with females, *P* < 0.001

**Table 2:** Puncture Max-angle (°)

	L1	L2	L3	L4	L5
Left	30.58 ± 7.13	34.23 ± 6.85	41.05 ± 6.10	45.39 ± 5.38	48.16 ± 6.88
Right	27.59 ± 7.70*	31.87 ± 8.52*	38.71 ± 7.31*	43.30 ± 5.95*	48.54 ± 6.89
Female	27.05 ± 6.93	36.01 ± 7.13	37.70 ± 6.85	42.76 ± 5.73	49.59 ± 6.01
Male	31.12 ± 7.64**	30.10 ± 7.34**	42.06 ± 6.07**	45.93 ± 5.36**	47.11 ± 7.45**
Mean	29.08 ± 7.56	33.06 ± 7.81	39.88 ± 6.83	44.34 ± 5.76	48.34 ± 6.88

*Compared with the left side, *P* < 0.001; ** Compared with females, *P* < 0.001

**Table 3:** Puncture success value

	L1	L2	L3	L4	L5
Left	40.51 ± 27.77	49.53 ± 23.82	61.59 ± 13.82	59.45 ± 11.07	48.42 ± 16.78
Right	22.55 ± 30.04*	34.77 ± 30.02*	50.82 ± 20.55*	52.95 ± 13.84*	47.59 ± 14.55
Female	25.39 ± 36.96	33.33 ± 31.06	51.70 ± 21.29	54.60 ± 14.81	50.97 ± 11.61
Male	37.67 ± 30.63**	50.53 ± 21.86**	60.60 ± 13.29**	57.80 ± 10.52**	45.04 ± 18.47**
Mean	31.53 ± 34.45	42.15 ± 28.06	56.21 ± 18.30	56.20 ± 12.93	48.01 ± 6.88

*Compared with the left side, P < 0.001; ** Compared with females, P < 0.05

**Table 4:** Puncture success rate

	L1	L2	L3	L4	L5
LSR	127/200	167/200	191/200	196/200	175/200
RSR	90/200*	121/200*	167/200*	185/200*	176/200
BSR	78/200	115/200	167/200	185/200	166/200
ESR	139/200	172/200	191/200	196/200	185/200
FSR	84/200	117/200	170/200	187/200	186/200
MSR	133/200**	171/200**	190/200**	194/200	165/200**

*Compared with the left side, P < 0.01; ** Compared with females, P < 0.001

LSR: Left Success Rate; RSR: Right Success Rate; BSR: the Puncture Success Values of both sides were larger than or equal to 34 for the identical vertebra), and the Either Side Success Rate (ESR: the Puncture Success Value of either side was larger than or equal to 34). The Female Success Rate (FSR) was calculated as follows: for example, if for the L1 level, a puncture was performed through both sides, then for 100 females, there were 200 punctures. If, of the 200 punctures, 84 were considered successful, then the FSR = 84/200). The Male Success Rate (MSR) is calculated as for the FSR)

### Differences in lumbar levels

Analysis of variance revealed statistically significant differences in the maximum puncture distance, maximum puncture angle, and puncture success value according to the lumbar level. Further pairwise comparisons were performed by the least significant difference method, and the results showed no significant difference in the puncture success value between L3 and L4 (P = 0.997). However, in the other pairwise comparisons, the differences in the maximum puncture distance, maximum puncture angle, and puncture success value were significantly different at different levels (P < 0.001). Table 4 shows that the bilateral success rates for L1, L2, L3, L4, and L5 were 39.0 %, 57.5 %, 83.5 %, 92.5 %, and 83.0 %, respectively. The either-side success rates were 69.5 %, 86.0 %, 95.5 %, 98.0 %, and 92.5 % for L1 to L5. There were significant differences in the bilateral and either-side success rates among the different lumbar segments (P < 0.001).

### Differences between left and right

The differences in the maximum puncture distance and maximum puncture angle between the left and right sides were analyzed at each vertebral segment using a paired t-test. For L3 and L4, the difference in the maximum puncture distance between the left and right sides was significant. However, there were no significant differences for the other vertebral segments. The statistical analysis showed that for L1 to L4, the left maximum puncture angle was significantly larger than the right. However, a significant difference was not demonstrated for L5.

The difference in the puncture success value between the left and right sides was consistent with the difference in the maximum puncture angle. Statistically, the left puncture success value was larger than the right for L1 to L4 and was nearly identical to that of the right side for L5. The puncture success rates of the right and left sides at each vertebral level were compared with the chi-squared test. The left success rates were 63.5 %. 83.5 %, 95.5 %, 98.0 %, and 87.5 % from L1 to L5. The right success rates were 45.0 %, 60.5 %, 83.5 %, 92.5 %, and 88.0 % from L1 to L5. The results of the statistical analysis were consistent with the puncture success value. For L1 to L4, a puncture through the left pedicle had a higher success rate than a puncture through the right pedicle. For L5, there was no obvious difference in the puncture success rate between the left and right sides.

### Differences between men and women

A puncture could not be performed at the maximum angle because of an iliac crest block in 41 (9 women, 32 men) of the 200 patients. The maximum puncture distance in men was significantly longer than that in women for L1 to L5. The maximum puncture angle in men was larger than that in women for L1 to L4; however, the result for L5 was the opposite.

The results of the statistical comparisons of the puncture success value and puncture success rate between men and women were consistent. The female success rates were 42.0 %, 58.5 %, 85.0 %, 83.5 %, and 93.0 %, and the male success rates were 66.5 %, 85.5 %, 95.0 %, 97.0 %, and 82.5 % from L1 to L5. The puncture success rate in men was much higher than that in women for L1 to L4. However, puncture was more likely to be successful for L5 in women. The differences in the puncture success value between men and women were similar to the differences in the puncture success rate. A detailed statistical analysis is presented in Table 4.

## Discussion

Unilateral PVP is attracting increasing attention because of its advantages, including a shorter surgery time, less trauma, less bone cement leakage, and less radiation exposure compared with bilateral PVP. PVP via the unilateral pedicular approach reportedly achieves an ideal clinical effect in the treatment of osteoporotic fractures [10, 13, 16–19].

However, there have been reports that the use of the bilateral approach during PVP might have better long-term outcomes and result in less cement leakage than unilateral puncture for patients with acute osteoporotic vertebral fracture. The puncture angle has been intentionally increased to improve the probability of puncture success; this might increase the risk of spinal cord and nerve root injury and bone cement leakage. It is difficult for surgeons to evaluate the pros and cons of each PVP approach [11, 12, 15]. Because of the anatomical distinctions among different lumbar levels and between the two sexes, the degree of difficulty in unilateral puncture is variable. Analysis of the differences in the unilateral puncture success rates among different lumbar segments, between the sexes, and between the left and right sides is relevant because it might guide spinal neurosurgeons to select the optimal PVP approach. In this study, we explored the anatomical differences among different lumbar levels through puncture simulation using MRI and provided reference points for the surgeons in selecting a unilateral or bilateral pedicular PVP approach.

In our study, the puncture success value, which was more objective and specific than the puncture success rate, was defined to assess the difficulty of unilateral puncture. The puncture success values for L1 and L2 were lower than those for the other levels. Particularly for L1, the mean puncture success value was <34, and it successful puncture appeared to be difficult at this level. However, the puncture success values for L3 and L4 were >34, and we consider that unilateral PVP is feasible and reliable for these segments. Although the pedicle diameter of L5 was the largest of the five vertebrae tested, the puncture success value was slightly lower than that for L3 or L4 because of the iliac crest block. Compared with that of L1 and L2, it was easier to succeed with a unilateral puncture. The puncture success rate and puncture success value were higher in men than in women except at L5. A higher incidence of iliac crest block in men led to a reduction of the puncture success rate. The data analysis revealed obvious differences in the puncture success rate and puncture success value between the left and right sides except at L5. For L1 to L4, the puncture success rate and puncture success value were higher on the left than on the right, which may have occurred because of anatomical differences between the left and right sides of the lumbar spine. The underlying cause remains to be further studied.

Studies by Steinmann et al. [11, 12, 14] on vertebral stiffness under different conditions of bone cement distribution suggested that with unipedicular PVP, biomechanical balance predominantly depends on the distribution of bone cement. If the bone cement is augmented exclusively on one side, the stiffness of the nonaugmented side might be significantly lower than that of the augmented side, which might lead to an imbalance of stress on the vertebrae. However, when the cement augmentation crossed the midline, the stiffness of both sides increased comparatively, and biomechanical balance was thus achieved.

In our study, the anterior trisection point was set as the target for successful puncture as in previous studies. Generally, bone cement can diffuse to the contralateral side, and biomechanical balance can be achieved if the puncture reaches the target. According to our study, a unilateral puncture cannot consistently reach the ideal target. The success rate of lumbar unilateral PVP via the pedicular approach is closely associated with the vertebral segment, patient sex, and left or right location. Given these findings, we present the following suggestions: For L1 and L2, particularly in women, it is reasonable and safe to select bilateral PVP. For L3 to L5, unilateral puncture is feasible and reliable, particularly for men. Success appears to be more likely with a puncture through the left pedicle for L1 to L4. Even for L4, for which unilateral puncture has a high probability of success, unilateral puncture failure remained a possibility in a few patients. It is necessary and beneficial to carefully analyze the imaging data before determining the optimal approach for each individual [13, 16].

These statistics are theoretical and should be used exclusively to illustrate the trends in puncture difficulty among different segments. Consistent performance of the puncture at the maximum angle cannot be ensured; therefore, the actual unilateral puncture success rate might be lower, which is one of the limitations of our study. The puncture success and safety are closely related to the operator’s experience and to the ancillary equipment. Extensive experience with the puncture procedure and effective assistance from the O-arm or C-arm could improve the puncture success rate.

This study had the following limitations. The anteroposterior and left–right diameters of the vertebral body and the pedicular width affected the success of unilateral puncture. Additionally, an iliac crest block should be considered for L5. These factors were considered to the maximum extent in our study design. However, the MRI scans were obtained parallel to the upper endplate. In practice, punctures parallel to the upper endplate might not be necessary in every case. The anatomical conformation of the pedicle in the coronal and sagittal planes also influenced the puncture.

Another limitation is that we defined Point T as the demarcation point of puncture success based on prior research. However, there is some controversy about the definition, and some researchers hypothesize that vertebrae can be strengthened bilaterally only if the puncture reaches or passes the midline of the vertebral body and the bone cement is distributed to the other side of the vertebral body [6, 7]. In previous studies, the bone cement diffused to the other side only if the puncture reached or approached the midline; thus, biomechanical balance was achieved.

## Conclusion

The success rate of lumbar unilateral PVP via the pedicular approach is closely associated with the vertebral segment, patient sex and left or right location. For L1 and L2, the performance of bilateral PVP is safe and reasonable, particularly for women. For L3 to L5, unilateral puncture is feasible and reliable, particularly for men. For L1 to L4, PVP appears to be more likely to succeed with puncture through the left pedicle.
